# A genotypic trade‐off between constitutive resistance to viral infection and host growth rate

**DOI:** 10.1111/evo.13623

**Published:** 2018-10-21

**Authors:** Lewis J. Bartlett, Lena Wilfert, Michael Boots

**Affiliations:** ^1^ Centre for Ecology and Conservation, College of Life and Environmental Sciences University of Exeter Penryn United Kingdom; ^2^ Department of Integrative Biology University of California Berkeley California 94720

**Keywords:** Evolution, host evolution, parasite, *Plodia interpunctella*, resistance, trade‐off

## Abstract

Genotypic trade‐offs are fundamental to the understanding of the evolution of life‐history traits. In particular, the evolution of optimal host defense and the maintenance of variation in defense against infectious disease is thought to be underpinned by such evolutionary trade‐offs. However, empirical demonstrations of these trade‐offs that satisfy the strict assumptions made by theoretical models are rare. Additionally, none of these trade‐offs have yet been shown to be robustly replicable using a variety of different experimental approaches to rule out confounding issues with particular experimental designs. Here, we use inbred isolines as a novel experimental approach to test whether a trade‐off between viral resistance and growth rate in *Plodia interpunctella*, previously demonstrated by multiple selection experiments, is robust and meets the strict criteria required to underpin theoretical work in this field. Critically, we demonstrate that this trade‐off is both genetic and constitutive. This finding helps support the large body of theory that relies on these assumptions, and makes this trade‐off for resistance unique in being replicated through multiple experimental approaches and definitively shown to be genetic and constitutive.

The understanding of trade‐offs remains central to evolutionary ecology (Shoval et al. 2012; Acerenza 2016). They are fundamental to understanding life histories in general (Roff and Fairbairn 2007), and more specifically in the context of the evolution of infectious disease, trade‐offs are generally assumed to determine both the evolution of pathogen virulence (May and Anderson 1983; Alizon et al. 2009; Alizon and Michalakis 2015), and host resistance to pathogens (Gillespie 1975; Sheldon and Verhulst 1996; Gemmill and Read 1998; Boots and Haraguchi 1999). In particular, the idea that defense against infectious disease is costly underpins a large theoretical and empirical literature examining the determinants of optimal defense (Boots and Bowers 1999; Boots and Haraguchi 1999; Gandon et al. 2002, 2008; Miller et al. 2007; Best et al. 2011; Best and Hoyle 2013; Gandon and Vale 2013) . Overall, the costs of resistance to pathogens are a well‐studied phenomenon (Antonovics and Thrall 1994; Brown 2002; Graham et al. 2005; Best et al. 2008; Schwenke et al. 2016). However, there is ambiguity in the nature of many trade‐offs demonstrated empirically and those modeled by theoreticians; specifically, whether trade‐offs are facultative or constitutive, and genetic or plastic (Reznick 1985; Roff and Fairbairn 2007; Cressler et al. 2015). Facultative costs are costs paid only when confronted with a pathogen, whereas constitutive costs are paid regardless of whether a pathogen is encountered. Genetic costs are directly inherited, and associated phenotype prevalences change only by evolution. Plastic phenotypes (such as those determined by epigenetics or maternal signaling) can vary in their prevalence without evolutionary changes in the population.

Costs of resistance are often measured when there is exposure to the pathogen (Dallas et al. 2016)–however, such measures represent activated facultative costs only, and cannot inform us about the costs paid by organisms that never encounter a pathogen. Such facultative costs of immune activation are now well demonstrated (Nordling et al. 1998; Moret and Schmid‐Hempel 2000; Armitage et al. 2003; Bonneaud et al. 2003; Sadd and Siva‐Jothy 2006); however, evolutionary costs of immunological maintenance in the absence of disease (constitutive costs), due to negative genetic correlations between immunocompetence and fitness, are typically assumed in the evolution of defense literature (e.g., Boots and Bowers 1999; Boots and Haraguchi 1999; Gandon et al. 2002, 2008; Miller et al. 2007; Best et al. 2011; Best and Hoyle 2013; Gandon and Vale 2013). The absence of empirical demonstrations of genetic, constitutive trade‐offs is a recognized problem (McKean et al. 2008; Cressler et al. 2015) as these are the costs to resistance that are modeled by theoreticians; empirical studies are rarely designed to show a trade‐off in such a way as to conclusively meet these strict criteria. Demonstrating trade‐offs that meet the criteria of being both constitutive and genetic is therefore a critical goal of bridging empirical and theoretical work on the evolution of host resistance to pathogens. These costs are important because they not only define optimal resistance but also because they are essential in explaining the wide‐spread variation apparent in the susceptibilities of hosts to pathogens (Bowers et al. 1994; Henter and Via 1995; Schmid‐Hempel 2003; Duffy and Forde 2009). Without clear evidence of whether a trade‐off is definitively genetic or plastic, and constitutive or facultative, it remains unsuitable for incorporation into theoretical models. Although one part of this solution is further work to build theoretical models on facultative and plastic costs, and to explore how these complementary routes to resistance interact, it is also critical to validate the current body of theory by showing trade‐offs that are definitively genetic and constitutive. Although in reality many different routes to resistance do all interact, theory tackling subsets of these route should be empirically integrated before being further developed.

Demonstrations of resistance costs in wild populations (Duffy et al. 2012; Auld et al. 2013; Susi and Laine 2015) and through selection experiments (Fuxa and Richter 1989; Boots and Begon 1993; Kraaijeveld and Godfray 1997; Luong and Polak 2007; Duncan et al. 2011) have provided evidence of constitutive costs of resistance in the absence of infection. However, these studies do not necessarily rule out the well‐documented phenomena of transgenerational immune‐priming or epigenetic (plastic) effects (Roth et al. 2009; Tidbury et al. 2011, 2012; Best et al. 2013) as such populations are not historically naïve to their pathogens. Studies have shown that resistance rapidly disappears in the absence of the pathogen (Fuxa and Richter 1989; Duncan et al. 2011), suggesting some cost to maintaining resistance, even if it is a phenotypically plastic or epigenetic effect. Examining these apparent trade‐offs with a plurality of experimental approaches will help to rule out some of the potential confounds of specific experimental designs and clarify whether trade‐offs are indeed demonstrably constitutive and genetic.

Here we take a new approach to examine a previously documented, highly cited evolutionary trade‐off in the moth *Plodia interpunctella* (Hübner). A resistance cost in this system was first demonstrated by Boots and Begon (1993), and a further selection experiment confirmed this trade‐off in a fully replicated experimental design (Boots 2011), making it one of the few evolutionary trade‐offs confirmed by multiple independent selection experiments. However, such selection experiments cannot rule out intergenerational effects. Here we used inbred isolines rather than selection experiments to gain novel insights into this evolutionary trade‐off and prove it meets the criteria detailed in McKean et al. (2008) and Cressler, Graham, and Day (2015) of being both genetic and constitutive. Genetically restricted lines have shown great value in their application to evolutionary biology in model species such as *Drosophila melanogaster* (David et al. 2005), and have been successfully used elsewhere to demonstrate variation in insect‐virus systems, for example, in vector competence (Jacobson and Kennedy 2013). Inbred isolines were established in this system to evolve to different genotypes via genetic drift during inbreeding, reflecting variation in the initial outbred stock population. When these genetically restricted isolines are assayed, this variation between isolines recovers the previously documented costs associated with resistance to a pathogen. This is despite all isoline populations being historically naïve to the pathogen (all evolution occurs in the absence of infection), providing the strongest evidence yet that the evolutionary trade‐off is based on genetic, constitutive costs of resistance.

## Materials and Methods

The host organism used was the pyralid moth *P. interpunctella*, the larvae of which are a common grain‐feeding pest of cereals worldwide (Mohandass et al. 2007). It has been used as a study species for a variety of biological experiments due to its ease of population maintenance and its importance as a food pest (Silhacek and Miller 1972; Mohandass et al. 2007). It exhibits a simple life history, divided into larval and adult stages. Eggs are laid into cereal media by adults in a semelparous event, larvae then develop in the food media until pupation, and following pupation adult moths emerge, mate, and a new generation of eggs are laid into the cereal medium (Gage 1995). Adults do not have functioning feeding physiology, and their reproductive success is broadly determined by their rate of development and pupal mass (Silhacek and Miller 1972; Boots and Begon 1993). *Plodia* larvae are susceptible to infection by the baculovirus *Plodia interpunctella* granulosis virus (PiGV; Sait et al. 1994). PiGV infections are obligately lethal, and infection occurs via oral consumption of viral occlusion bodies. PiGV has strong effects on *Plodia* population dynamics (Sait et al. 1994), and *Plodia* may evolve resistance to the virus at the cost of increased development time (Boots and Begon 1993, 1994; Boots 2011). Intrahemocoelic antiviral activity has been demonstrated (Saejeng et al. 2011), although this resistance is not connected to the often‐studied phenoloxidase pathway (Saejeng et al. 2010). Resistance is thought to occur mostly at the gut wall, through mechanical barriers such as the peritrophic membrane and apoptosis of infected gut wall cells (Begon et al. 1993; Tidbury 2012).

*Plodia* were raised following well‐established protocols. All lines originated from an outbred laboratory stock population of *Plodia*. Male‐female pairs were selected at their fifth larval instar, when males can be differentiated from females by the conspicuous gonads, visible as a dark spot on the back of the larva. To establish genetically isolated lines, single male‐female pairs were then placed in 250‐mL straight‐side wide‐mouth Nalgene jars (ThermoFisher Scientific, UK) with 20 g of food media. Food media was prepared in batches consisting of 250 g “Ready Brek” (Weetabix Ltd., UK), 150 g wheat bran (Bob's Red Mill), 100 g rice flour (Bob's Red Mill), 100 g brewer's yeast (MP Biomedicals), 125 mL glycerol (VWR), 125 mL clear organic honey (Dutch Gold Honey Inc.), 2.2 g methyl paraben (VWR), and 2.2 g sorbic acid (Spectrum Chemicals). Food media batches were homogenized with industrial mixers before being sealed and frozen for a minimum of 24 h prior to thawing at ambient temperature for use as growth media.

*Plodia* pairs were incubated at constant conditions of 27 ± 2^o^C and 35 ± 5% humidity, with 16:8 hr light:dark cycles. After four weeks, *Plodia* pairs had typically undergone a full generation cycle, where the founding pair had successfully pupated, eclosed, mated, laid eggs, and died, with progeny developing to their fifth instar in the supplied food media. For each line of full‐sibling progeny, full‐sibling male‐female pairs were placed as above in a new jar of food media. The subsequent generation represented one generation of full‐sibling inbreeding. For each line, five replicates were set up each generation. This was done as some pairs invariably produce no offspring due to a failure, or mismatch in timing, of pupation, eclosure, or mating. In cases were multiple replicates produced successful subsequent generations, one jar of full‐sibling larvae was randomly selected to found the next generation. This inbreeding regime was maintained for 30 generations to establish genetic isolines with very high levels of homozygosity (Rumball et al. 1994). Of the initial 25 founding lines, 20 lines became extinct in the first eight generations of inbreeding, likely due to five mating‐jar replicates being an inadequate insurance against unsuccessful mating during this period of intense inbreeding depression. The remaining five survived all 30 generations of inbreeding to establish the genetic isolines used in this study.

The growth rate of each isoline was estimated using two measures: time to pupation (development time) and pupal mass. For each isoline, 60 adult *Plodia*, known to have eclosed in the last 24 hr, were taken and placed on 200 g of food media in 1000 mL straight‐side wide‐mouth Nalgene jars (ThermoFisher Scientific) and incubated as above. After 11 days, 50 larvae on the first day of their third instar were taken from each isoline population and placed into individual compartments on 25‐cell compartmentalized square Petri dishes (ThermoFisher Scientific) (two Petri dishes per isoline). Each compartment contained ample food media. First‐day third‐instar larvae were identified by examining the size of their head (which changes only during molting and identifies different instars) compared to the size of their body (which if smaller in diameter than the head signifies their first day at that instar). Petri dishes were then incubated as above and checked daily to monitor larval development. All growth rate assays were set up simultaneously on the same day and incubated on the same shelf of the same incubator. The date of each larva's pupation was recorded, and two days later the pupa was extracted from its silk cocoon and weighed using a 1‐μg precision microbalance. Not all larvae were recovered, as some inevitably die due to handling or other causes of stochastic mortality ($\bar{n}$ = 18 larvae recovered per line, Supporting Information 2).

The susceptibility to pathogens of each isoline was characterized by comparing infection rates of larvae to PiGV. For each isoline, 300 first‐day third‐instar larvae were selected as described above. Larval cohorts of 50 larvae were placed in circular Petri dishes (six cohorts per isoline) and starved for 1 hr. Droplets of virus solution were then pipetted into these Petri dishes, with each cohort given one of six solutions. Virus solutions represented six doses: a control dose (no virus) and five doses of virus solution each diluted by an order of magnitude (such that the strongest dose is 10^4^ times stronger than the weakest). Solutions were diluted with distilled water, and otherwise made up to contain 0.1% Coomassie Brilliant Blue R‐250 dye (ThermoFisher Scientific), and 2% sucrose (ThermoFisher Scientific). Larvae voluntarily fed on the solution droplets due to the sucrose content, and were considered dosed when 50% of their alimentary track was stained blue (visible due to the blue dye and translucent larval body) at which point they were removed from the Petri dish. Twenty‐five suitably dosed larvae from each cohort were taken and placed individually in 25‐cell compartmentalized square Petri dishes and incubated for 10 days as above. All susceptibility assays were set‐up simultaneously on the same day and incubated on the same shelf of the same incubator. After 10 days, Petri dishes were frozen to kill all remaining live larvae, before being opened for counting. Infected larvae are easily recognized by their bright white cadavers, caused by the accumulation of viral occlusion bodies in the hemolymph. Uninfected larvae were easily identified as healthy larval cadavers or as developing pupae. Not all larvae were recovered to be categorized as either infected or uninfected, as some inevitably die due to handling or other causes of stochastic mortality ($\bar{n}$ = 18 larvae recovered per line per dose, Supporting Information 1).

All statistical analyses were undertaken using R (version 3.3.2 “Sincere Pumpkin Patch”). Differences between isoline growth rates were examined using ANOVAs, where tests were undertaken comparing mean pupal mass, mean development time, and mean growth rate (pupal mass/development time). Susceptibility was analyzed using generalized linear models (glms), with a binomial error structure and logit link function, where the response variable was whether each recovered larva was infected (1) versus uninfected (0) for each dose and line. Doses were analyzed after being transformed using a log_10_( *x* + 1) function. We compared models using ANOVA (chi‐squared) tests to eliminate model terms in the style of a backward stepwise model simplification (Crawley 2012). The starting (most complicated) model included the following explanatory terms: isoline, viral dose strength, and an interaction between the two (to test for heterogeneity in dose response). Significantly differing components of growth rate and susceptibility across the isolines were then tested for correlation against one another.

## Results

**Figure 1:**
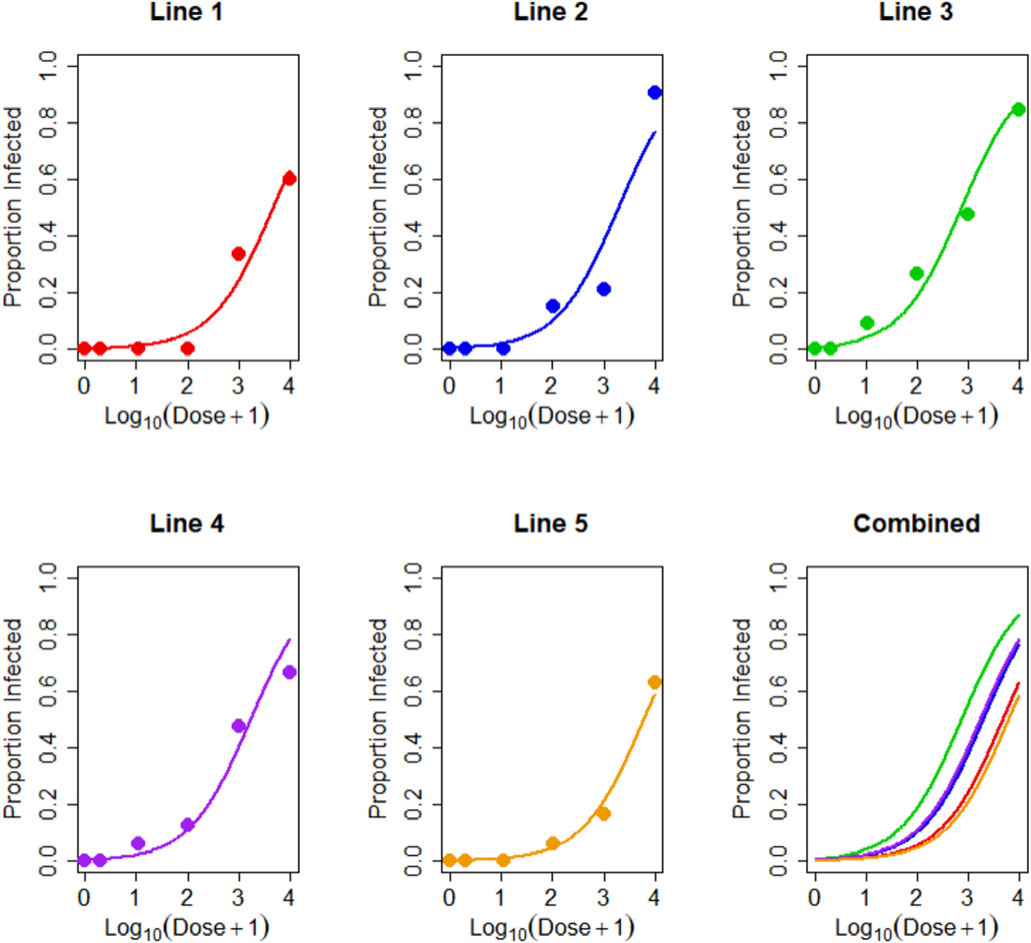
Plots showing susceptibility data and best‐model fit for each of the five isolines. Data are shown as the proportion of larvae that succumb to infection across the six doses, with doses plotted in logit space. Data and model fits for each line are shown in single panels and model fits are compared in the “Combined” panel. Differences between lines’ susceptibilities to the pathogen are clear from the distance between the plotted lines, with line 3 the most susceptible and line 5 the least. No fitted lines cross when plotted on the same axes, as would be expected when no significant interaction effect was apparent.

**Figure 2:**
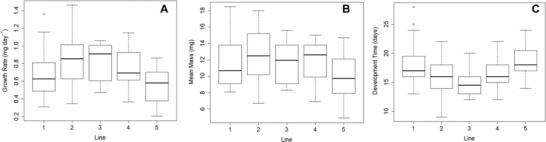
Plots comparing growth rate, mean mass, and development time of each of the five isolines. Growth rate (A) is calculated for each individual larva as the pupal mass (B) divided by the development time (C). Significant differences between isoline growth rates (A) and development time (C) were apparent, however no significant difference between lines was found for mean mass (B). Line 3 is the fastest growing, owing to its shorter development time, whereas line 5 appears to be the slowest growing.

Isolines differed in both their susceptibility to the pathogen and their development patterns (Figs. 1, 2). Growth rate (pupal mass/development time) significantly differed across the five isolines (*F*_4,79_ = 2.86, *P* = 0.029; Fig. 2A), however this was driven principally by a significant difference in development time between lines (*F*_4,79_ = 2.86, *P* = 0.0054; Fig. 2C), with no detectable significant difference in pupal mass (*F*_4,79_ = 2.86, *P* = 0.18; Fig. 2B). As expected, higher dose strengths lead to higher infection likelihoods (*P* < 0.001). There was no evidence for heterogeneity in dose response between lines (Fig. 1) when modeled as an interaction effect between dose and line (*P* = 0.397), however lines did differ in their susceptibility to infection overall (*P* = 0.0057).

**Figure 3:**
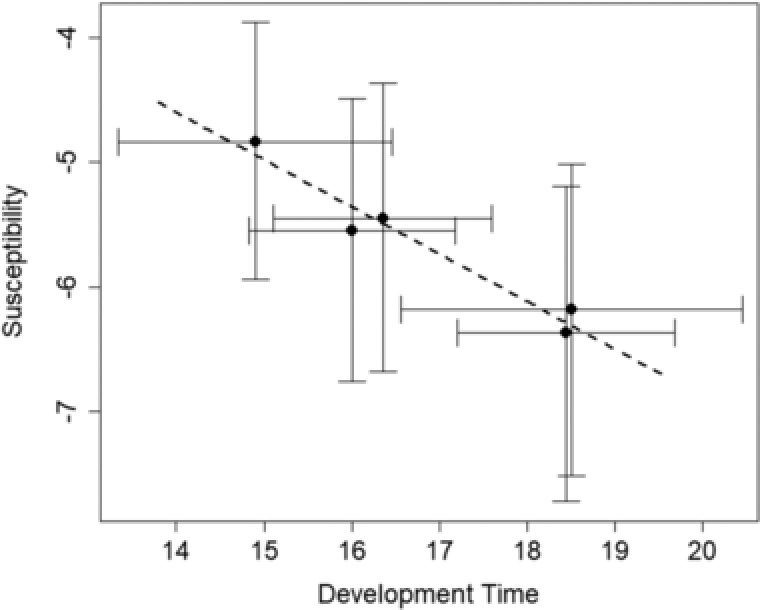
Plot showing correlation between susceptibility (y‐axis) and development time (x‐axis) for the five isolines. 95% confidence intervals are plotted around the point estimates for both susceptibility and development time. Susceptibility estimates were extracted from the binomial glm used to analyze the infection data (Fig. 1). Development time (Fig. 2C) was directly measured as described in the manuscript. The correlation was found to be significant using a Pearson's product moment correlation test. Line 3 is the most susceptible and has the shortest development time; the least susceptible lines with the longest development times are lines 1 and 5.

Given the lack of heterogeneity in dose response between lines’ susceptibilities, each line's susceptibility could be characterized using the line‐effect values extracted from the binomial glm. These extracted values were directly tested (without transformation back to identity space) for correlation against the five lines’ demonstrably different mean development times using a Pearson's product moment correlation. The two isoline characteristics showed a significant correlation (*r*_3_ = –0.976, *P* = 0.004), where a faster development time correlated with greater susceptibility to the pathogen (Fig. 3).

## Discussion

Our results demonstrate a trade‐off between development time and resistance to a viral pathogen (Fig. 3). This is not the first time such a trade‐off has been shown in this system. Previous experiments (Boots and Begon 1993; Boots 2011) have shown that populations of *Plodia* can be selected to evolve increased resistance to PiGV at the cost of longer developmental times. We confirm the existence of this trade‐off between resistance and development time using an alternative method, without selection for specific traits. For practical reasons it is often the case that key findings in evolutionary ecology are never replicated. Given the multiple methods now used to demonstrate this evolutionary trade‐off in at least three separate experimental instances, the cost of resistance in *Plodia* is arguably one of the most robust and well‐evidenced evolutionary trade‐offs in evolutionary ecology.

This study's use of inbred isolines provides novel insights into this trade‐off, with important ecological and evolutionary implications. Unlike in previous selection experiments, all sampling in this study was carried out using host populations that were not historically exposed to the pathogen. Evolution of each isoline to a genotype with either higher or lower resistances was achieved through inbreeding and genetic drift, rather than selection for particular traits, and all evolution occurred in absence of any infection. Although previous experiments have taken steps to try and rule out intergenerational effects by using larvae two generations removed from the selective pressure, it remained possible that epigenetic effects were underpinning the apparent trade‐off. This study rules out such possible confounds, and confirms that this widely cited evolutionary trade‐off is genetic in its basis.

Similarly, this study also demonstrates that longer development time is a constitutive cost of host resistance, rather than facultative. Interestingly, resistance to *Bacillus thuringiensis* (Bt) toxin has also been linked to longer development times in *P. interpunctella* (Oppert et al. 2000). Constitutive costs of resistance are understood to be more difficult to detect empirically (Armitage et al. 2003; Cressler et al. 2015), but have now been demonstrated in multiple ways in this system. This experiment shows that the cost of resistance (longer development time) manifests even in larvae that are evolutionarily naïve to, and have not been exposed to, the pathogen. This differentiates the cost of resistance in this system from cases where resistance costs manifest only after exposure to a pathogen (Dallas et al. 2016). The importance of demonstrating that this evolutionary trade‐off is underpinned by a genetic, constitutive cost begins to help validate the rich body of theory on the evolution of host resistance to pathogens. As an evolutionary force, genetic resistance to pathogens is critical in theories from the active evolutionary management of our agricultural food supply (Brown 2002; Seifi et al. 2013), to the evolution of sexual reproduction (Hamilton et al. 1990; Ashby and King 2015) especially regarding the well‐supported Red Queen hypothesis (Morran et al. 2011), which relies specifically on genetically underpinned resistance. Ongoing research in this field reveals many nuances concerning costs of resistance such as how they vary with diverse environmental factors (Boots and Begon 1994; Lazzaro and Little 2009; Boots 2011; Ferris and Best 2018), or how differences in types of resistance or immunity may have different evolutionary outcomes, interacting further with the consequences of infection (e.g., morbidity or sterility; Ferguson et al. 2001; Best et al. 2009; Donnelly et al. 2015). Still, the evolutionary importance of these resistance costs may be even greater than previously thought, as resistance to multiple pathogens may carry increasingly accelerating costs to the host (Koskella et al. 2011). Empirically validating model assumptions, as is done in this study, is therefore critical to the advancement of this field of evolutionary research.

Methodologically, this study confirms that strict inbreeding regimes are capable of yielding experimentally useful isolines in *Plodia*. Beginning with randomly selected individuals of an outbred laboratory stock population, highly homozygous isolines can be established with genotypes that differ in competitively relevant traits. Indirectly, this also validates that “outbred” laboratory stock populations of *P. interpunctella* do maintain appreciable amounts of standing genetic variation. There is potential for this technique to be used widely in this system, including to improve on the results shown here. In particular, beyond the simple existence of evolutionary trade‐offs between host resistance and other competitive traits, the shape of such trade‐offs is under important scrutiny. This shape of resistance‐cost trade‐offs has crucial evolutionary relevance (Boots and Haraguchi 1999; de Mazancourt and Dieckmann 2004; Bowers et al. 2005; Hoyle et al. 2008; Boots et al. 2009), with qualitatively different evolutionary outcomes depending on trade‐off shape. Inferences have been made about the shape of the trade‐off in this *Plodia*‐PiGV system before, implying a decreasing costs trade‐off shape (Mealor and Boots 2005), where increasing investment in resistance yields increasingly higher gains per unit of investment, rather than a trade‐off shape where investment in resistance yields large benefits at low resistance levels but much smaller increases in resistance when resistance is already high. Despite the importance of these differences, direct characterization of resistance‐cost trade‐offs has yet to be achieved in any comparable system. With a larger number of inbred isolines examined, it may be possible to use this technique to examine directly what the shape of the resistance‐cost trade‐off is in this system. In this study, too few isolines survived the inbreeding regime, and confidence intervals around susceptibility and development time were too large, for such precise analysis of the apparent trade‐off. We speculate that with a greater capacity for mating‐jar replicates, more lines would have survived the initial period of intense inbreeding depression, and that the loss of most of these lines was principally stochastic due to overall very low mating success during this stage of the experiment.

Plastic adjustment of development time has been demonstrated in *Plodia* (Gage 1995), a phenomenon that may play a part in other aspects of host defense. By using highly homozygous isoline genotypes in this study to examine a genetic, constitutive component of host resistance we eliminate the possibility of plastic effects. But applications in understanding plasticity of developmental and susceptibility traits is also equally possible, where eliminating genetic “noise” in data (via the use of homozygous isolines) is a powerful study tool. Given the aforementioned need to work toward understanding the interacting, complementary routes of resistance organisms exploit, use of such isolines to study both genetic and plastic, and facultative and constitutive, costs to resistance may prove useful for building future theoretical syntheses. Additionally, such inbred isolines can be used as a standardized host background for studies seeking to better understand pathogen evolution. This system has been used to demonstrate selective pressures on the adaptation of PiGV before (Boots and Mealor 2007); similar experiments may benefit from the use of inbred host isolines when characterizing viral traits.

Our approach of using inbred lines has yielded clearly beneficial insights into this evolutionary trade‐off, however the very high rates of population loss during severe inbreeding (80% lost during first eight generations, with no further losses after this initial stage) is worth some consideration. Following the period of severe inbreeding depression, the purging of strongly deleterious recessive alleles, the surviving inbred lines should be highly homozygous with no lethal or sterility‐inducing gene variants. The inbred lines here therefore should represent populations with almost no recessive lethal or recessive sterile alleles. Additionally, these lines are more likely to be highly viable, high‐fitness populations with lower prevalences of mildly deleterious genetic material. As to how this relates to nature is open to interpretation. Natural populations will likely contain far more recessive deleterious alleles evading selection than the inbred lines here, although the effect of this absence on our results is not clear. Outbred laboratory stocks are by design exposed to very benign environments with minimal selective pressure, which contrasts populations in nature. Laboratory stocks are therefore likely to accumulate far more deleterious alleles than are present in wild populations, and thusly be of lower fitness if reintroduced to a “natural” environment (Bryant and Reed 1999). Inbreeding may help reverse this by favoring highly viable, fit lines, which would better reflect natural populations under constant selection. Detailed discussion of the benefits and caveats of studying equivalent *Drosophila melanogaster* isofemale (inbred) lines is given in David et al. (2005).

In summary, this study confirms the previously evidenced trade‐off between resistance to a virus and development time in *P. interpunctella*, employing a method that avoids the confounds of selection experiments and proves that this trade‐off is genetic and constitutive. Additionally, this study demonstrates that inbred isolines of *P. interpunctella* can be established such that isoline genotypes differ in their traits due to genetic drift during inbreeding. These isolines may be a powerful tool for this system in further work examining the shape of the resistance‐cost trade‐off, and may also prove useful in experiments examining viral evolution in this system.

Associate Editor: K. King

Handling Editor: P. Tiffin

## Supporting information

Supplementary Information

Supplementary Information

Supplementary Information

## Data Availability

All data used in this study are made available in the Supporting Information.
